# Interaction of Non-Specific Lipid-Transfer Proteins With Plant-Derived Lipids and Its Impact on Allergic Sensitization

**DOI:** 10.3389/fimmu.2018.01389

**Published:** 2018-06-20

**Authors:** Stephan Scheurer, Stefan Schülke

**Affiliations:** Molecular Allergology, Paul-Ehrlich-Institut, Langen, Germany

**Keywords:** non-specific lipid-transfer protein, allergen, lipid, fatty acid, (glycero)phospholipid, CD1d, invariant natural killer T cell

## Abstract

Non-specific lipid-transfer proteins (nsLTPs) represent a family of ubiquitous plant proteins belonging to the prolamin superfamily. nsLTPs are characterized by a globular α-helical structure stabilized by four disulfide bonds and a hydrophobic cavity which acts as ligand-binding site for a broad spectrum of lipids and hydrophobic molecules. nsLTPs are involved in membrane biogenesis and in the adaption of plants to abiotic and biotic stress. They display antimicrobial activity by the ability to permeabilize the cell membrane of phytopathogens. Moreover, in the presence of lipids, nsLTPs are suggested to activate the plant immune system by a receptor-dependent mechanism. Additionally, nsLTPs from pollen and plant-derived food, in particular type 1 nsLTPs (9 kDa), are described as potent allergens. Within the nsLTP family Pru p 3 from peach is the clinically most relevant allergen which can cause genuine food allergy and frequently elicits severe clinical reactions. So far, the allergenic properties of nsLTPs are attributed to both their low molecular mass and their high thermal and proteolytic stability which allow them to reach the immune system in a biological intact form. Recently, the interaction of nsLTPs with lipids has been suggested to increase their allergenic properties and to promote the allergic sensitization to these proteins. This review will summarize the current knowledge on diversity of lipid ligands of plant LTPs, and illustrate recent studies performed with allergenic nsLTPs to investigate the effect of lipid binding on the structural modification and IgE-binding properties of proteins, and finally the potential effect on the innate immune responses.

## Introduction

The incidence of allergies is increasing worldwide (1, 2). However, the reason why only few protein families cause aero- and food allergy is still unsolved. There is increasing evidence that the allergenicity of proteins cannot be solely attributed to structural and physicochemical properties of the molecules itself (3). Factors which determine the allergenicity of food proteins are highly diverse, comprising also matrix effects, and the interaction of allergens with natural ligands affecting their antigenicity and immunogenicity (3, 4). Upon ingestion of food, allergens and lipids, including fatty acids (FAs), glycerolipids [triglycerides and phospholipids, e.g., phosphatidylcholine (PC)], or sphingolipids, are delivered to the immune system, either admixed in an unbound form or as a complex when lipophilic molecules act as natural ligands of allergens. In both cases, the presence of lipids was suggested to modify the allergenicity and the immune-modulating properties of the antigens (5).

## Impact of Lipids on the Sensitization Capacity of Food Allergens

Several studies indicated lipid mediators derived from bacterial and pollen contaminants and dietary lipids to modulate the allergenic properties of proteins (6). Phospholipid binding to Ber e 1 (major Brazil nut allergen, 2S albumin) *via* the engagement of IL-4-producing invariant (i)NKT cells (7, 8), binding of dietary medium-chain triglycerides to peanut proteins, PC binding to the Bet v 1-homologous apple allergen Mal d 1 (9), phosphatidylglycerol (PG) binding to Ara h 1 (7S globulin from peanut) and Sin a 2 (11S globulin from mustard) (10), and preincubation of peanut extract and allergen Ana o 2 from cashew with oleic acid (11) were reported to change the immunogenicity of the respective allergens by either increasing allergen stability and IgE-reactivity or stimulating allergen absorption and subsequent T_H_2 responses. Here, lipid extracts from nuts favor allergen-induced inflammatory responses by increasing IL-4/IL-10 ratios and IL-1β secretion from human monocyte-derived DCs (moDCs) (11). Moreover, purified peanut-derived lipids applied together with either Arh 1 or Ara h 2 were reported to trigger pro-inflammatory responses (IL-8, IL-6, and TNF-α secretion) while inhibiting anti-inflammatory IL-10 release from human keratinocytes (12).

In summary, lipophilic components, mainly the oil or lipid fraction from nuts, have immune-modulating capacity and can affect the allergic response. However, in contrast to the mode of the molecular interaction, (1) the impact of lipids on the allergic sensitization and (2) the effect of lipid binding to food allergens on their immunogenicity and allergenicity are less investigated. Lipid-binding properties have been suggested for numerous food allergens, e.g., the Bet v 1-family (Ara h 8, peanut, Mal d 1, apple), lipocalins (Bos d 5, bovine milk), 2S albumins (Ber a 1, brazil nut, Sin a 1, mustard), 7S and 11S globulins (Ara h 1 and Sin a 2), alpha-lactalbumin Bos d 4, vicilin Gly m 5 (soybean), legumin-like protein Ana o 2 (cashew), as well as plant non-specific lipid-transfer proteins (nsLTPs) (5, 6). From an allergological viewpoint, the interaction of lipids with nsLTPs is of special interest because these proteins frequently induce severe clinical symptoms. Therefore, this review will focus on the interaction of different lipids with nsLTPs.

## Structural Interaction of Food-Derived Lipids with nsLTPs

### Biological Properties of nsLTPs

Non-specific lipid transfer proteins represent a family of small and ubiquitously expressed plant proteins belonging to the prolamin superfamily. nsLTPs are cysteine-rich proteins which are stabilized by four internal disulfide bonds. Two nsLTP subfamilies, 9 kDa nsLTP1 and 7 kDa nsLTP2, are known, both characterized by a globular α-helical structure with a tunnel-like hydrophobic cavity. This cavity makes them suitable for binding and transportation of various lipids (13, 14). Although the physiological function of nsLTPs is contentious, strong evidence has been provided for nsLTPs to function as intra- and extracellular carriers for a broad spectrum of lipids required for membrane biogenesis. Furthermore, nsLTPs are members of the family of pathogenesis-related proteins 14 and are involved in the adaption of plants to abiotic and biotic stress. The role of nsLTPs in plant defense is suggested to result from (1) a direct antimicrobial activity facilitated by permeabilization of cell membranes of phytopathogenic bacteria and fungi and (2) a putative fungal elicitin (nsLTP homolog) receptor-dependent activation of the plant immune system in the presence of lipids (15–18). By contrast, several reports suggest that lipid-binding and antimicrobial properties are spatially separated (19).

### Interaction of nsLTPs With Lipids

The majority of studies investigating the structural interaction of lipid ligands with plant nsLTPs has been performed in monocotyledons, e.g., barley, rice, wheat, or maize, to elucidate their physiological function (17). nsLTPs can bind a wide range of ligands, including molecules of organic solvents, certain drugs, acyl derivatives of coenzyme A, sterols, prostaglandin B2 (PGB_2_), and likely most importantly aliphatic lipids, comprising glycerophospholipids (PG and PC) and derivatives thereof, including lysophospholipids (lyso-PG and lyso-PC), as well as FAs or FA-dervatives (14, 20). Plant nsLTPs can bind one or two fatty acyl chains by non-cooperative or cooperative binding sites with K_D_s in the low micromolar range: ^1^H NMR and 2.1-Å crystal structure analysis revealed that TaLTP1.1 from wheat (*Triticum aestivum*) can accommodate either one molecule of 1,2-dimyristoyl-PG or two molecules of 1-myristoyl-2-hydroxy-PC (L-α-myristoyl-PC, lyso-PC, LMPC) in a “head-to-tail” orientation (14, 20). In addition, TaLTP1.1 was crystallized in a complex with PGB_2_, an FA derivative originated from C_20_-unsaturated arachidonic acid. Moreover, studies provided evidence that nsLTPs form the most stable complexes with different C10–C18 chain unsaturated FAs containing one or two double bonds in the *cis* configuration, e.g., linoleic and oleic acid (18). Using pea nsLTP1 it was reported that unsaturated FA, e.g., linoleic (C18:2, all-*cis*-9,12) and linolenic (C18:3, all-*cis*-9,12,15) acids rather than saturated FAs, and lysolipids, e.g., negatively charged LMPG (C14) and L-α-palmitoyl-phosphatidylglycerol (LPPG) (C16), showed strongest interaction with the allergen (21). Notably a tomato LTP was found to bind L-α-palmitoyl-lysophosphatidylcholine (C16). This complex was stable even after thermal treatments with up to 105°C (22).

### Structural Modification Induced by Lipid Binding

Of note, nsLTPs lack a marked specificity for ligands which can be attributed to the flexibility of the van der Waals volume of internal hydrophobic cavities sufficient to accommodate either single- or double-chain lipids. The structural variability of the internal cavity has been shown by a more than fourfold increase of the cavity volume after binding of FAs (18). The volume of the cavity of TaLTP1.1 increases from 300 ± 50 to 786 ± 43 Å^3^ upon PGB_2_-binding (14). Lipid binding to nsLTPs by the engagement of conserved amino acids at the entrance of the tunnel and subsequent reshaping of the internal hydrophobic cavity is accompanied by a conformational (micro)heterogeneity of the molecules. So far, it is unclear whether the conformational modification affects biological function and allergenicity of the lipid-complexed nsLTPs.

### Lipid Binding to nsLTP1 and nsLTP2

Several studies demonstrated the heterogeneity of lipid interaction with nsLTPs from different plant species, between nsLTP isoforms, or members of the nsLTP1 and nsLTP2 subfamilies. However, it seems that the highly conserved amino acids Arg_44_ and Tyr_79_ in nsLTP1 (e.g., from rice) and Phe_36_, Tyr_45_, and Tyr_48_ in nsLTP2 at the entrance of the hydrophobic cavity are crucial for lipid “uptake” (18). Hence, these data suggest that all nsLTPs are capable to bind lipids, but the specificity and binding affinity of the ligands cannot be deduced. Furthermore, wheat nsLTP1 (TaLTP1.1) has been shown to bind LMPC (PDB 2BWO) (20), whereas a crystal structure showed wheat nsLTP2 (TaLTP2.1) to interact with LPPG (23). In addition, nsLTP1 has been reported to accommodate either one or two molecules of linear mono- or diacylated lipids, whereas nsLTP2 can bind even planar sterol molecules (19). The binding properties of nsLTPs were attributed to the size of the hydrophobic cavity being more spacious for nsLTP2. Another study showed the cavity of rice nsLTP2, although it is smaller than that of rice nsLTP1, to be flexible enough to accommodate the voluminous sterol molecule (24).

In summary, the structural interaction between lipid ligands and nsLTPs shows the diversity of bound ligands and the heterogeneity of binding modalities. However, the question remains whether the type and mode of lipid binding determines the biological function and whether it affects the allergenic properties of nsLTPs.

## Interaction of Lipids with Allergenic Food nsLTPs likely Effects the Stability and IgE-Reactivity of the Complexed Allergen

Plant-derived nsLTPs are among the clinically most important food allergens frequently eliciting severe clinical reactions (25, 26). Curiously, nsLTPs are described as allergens in the Mediterranean area rather than in Central and Northern Europe. Actually, more than 35 nsLTPs, almost all belonging to the nsLTP1 subfamily, have been described as food allergens (according the IUIS allergen nomenclature subcommittee) (26). So far, the strong allergenic properties of nsLTPs are attributed to their high resistance to both heat treatment and gastrointestinal enzymes (26). However, up to now only a limited number of studies investigated the interaction between lipids and allergenic nsLTPs and its effects on IgE-binding capacity and allergenic potency.

Peach nsLTP Pru p 3, the prototypic member of the nsLTP family, has been described as a genuine allergen inducing primary sensitization (26). *In vitro* Pru p 3 was able to cross Caco 2 monolayers (27). The transcellular transport of Pru p 3 occurred *via* a lipid raft pathway that did not disturb the integrity of the tight junctions but induced epithelial cell-derived production of the T_H_2-promoting cytokines TSLP, IL-33, and IL-25 (27).

The hydrophobic cavity of Pru p 3 displays a remarkable degree of plasticity allowing for the binding of ligands in different orientations (25). From structural analysis, the authors assumed a hypothetical ligand bound to Pru p 3 resembling lauric acid. *In silico* analysis revealed the amino acid residues supposed to participate in lipid binding are conserved in the allergenic peach nsLTP Pru p 3 (21).

Notably linoleic, palmitic, and oleic acids are described as main components of peach oil (28), and linoleic acid has been found as major FA in wheat (29). Recently, substantial binding of palmitic (and linoleic) acid to Pru p 3 rather than to wheat LTP was demonstrated experimentally (29). Surprisingly, lipid binding to wheat LTP was associated with an increased susceptibility to gastrointestinal proteolysis, which was due to a conformational change and the exposure of an additional protease cleavage site (29). Nevertheless, the authors speculated that lipid binding to other nsLTPs may have an opposite effect by increasing the stability and therefore may be capable to contribute to the allergenicity of the molecules/respective allergens. In line with this, Vassilopoulou and co-workers showed a slightly protective effect to the gastric digestion of PC admixed with grape LTP (30). However, the protease-treated nsLTP:lipid complex did not show an increased allergenicity.

Of note, unsaturated FA (oleic, linoleic, and elaidic acid) and short chain saturated lauric acid (C12) were confirmed to bind to Pru p 3 by 1,8-ANS-competition assays (31). Binding of oleic but not stearic acid (saturated C18) increased IgE-reactivity and basophil activation by conformational changes. This effect was less pronounced for allergenic nsLTPs Jug r 3 (English walnut) and Cor a 8 (hazelnut) (32).

An independent study suggested a derivative of 10-OH-camptothecin potentially bound to the hydrophobic tail of phytosphingosine (C18) as the natural ligand for Pru p 3 (33). Both molecules, identified by ESI-qToF, were co-purified together with natural Pru p 3 (33). *In silico* analysis suggested phytosphingosine to be bound to the hydrophobic cavity of Pru p 3. Here, ligand binding was associated with a slight structural modification of Pru p 3 and a distortion of the CD spectrum of Pru p 3 in the 190–200 nm region (33).

In summary, several FAs, likewise saturated lauric acid and unsaturated oleic acid (31) as well as palmitic acid (29), and phytosphingosine (33) were described as ligands for Pru p 3 (Table 1). The conjugation of lipids to nsLTPs may influence the IgE-binding capacity by structural modification of allergens (31). So far, the effect of lipids on the stability of allergens is discussed controversially.

**Table 1:** Lipophilic ligands of food-derived nsLTPs.

Fatty acid	Formula	nsLTP	Reference
Decanonic acid [C10]	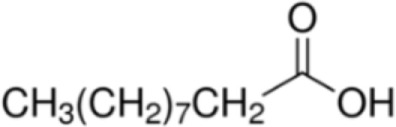	**Maize LTP**	(14)

Lauric acid [C12]	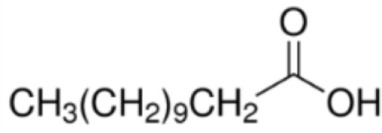	**Pru p 3** **Lentil LTP** Maize LTP	(29) (14)

Myristic acid [C14]	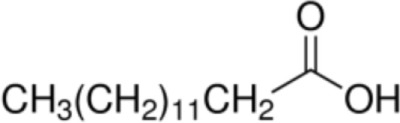	**Maize LTP** **Rice LTP**	(14)

Palmitic acid [C16]	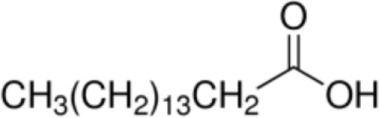	**Pru p 3** (Tri a 14) Maize LTP Barley LTP	(31) (14) (14)

Stearic acid [C18]	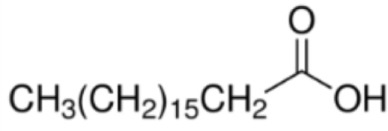	(Pru p 3) (Jug r 3) (Cor a 8) Maize LTP Rice LTP	(31, 32)

Palmitoleic acid [C16:1, cis-9]	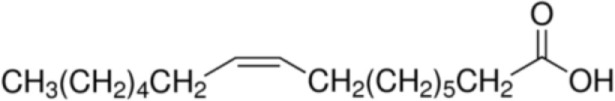	(Pea LTP) Maize LTP Rice LTP	(21)

Oleic acid [C18:1, cis-9]	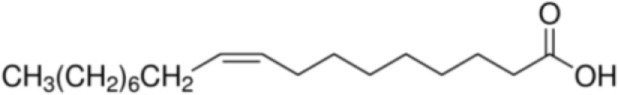	**Prup3** (Jug r 3) (Cor a 8) (Mal d 3) Maize LTP	(31, 32)

Ricinoleic acid [C18:1, cis-9, 12-OH]	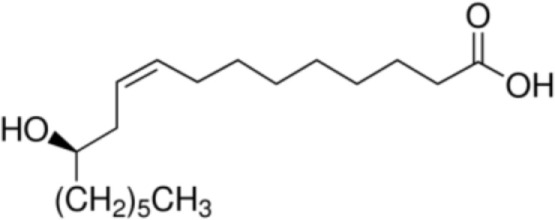	**Maize LTP**	(14)

Elaidic acid [C18:1, trans-9]	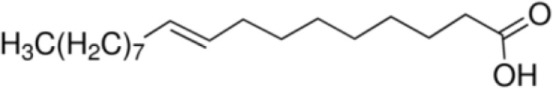	**Pru p 3**	(31)

Linoleic acid [C18:2, cis-9,12]	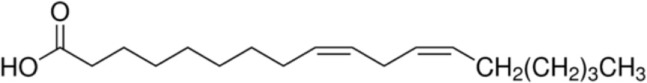	**Prup 3** **Pea LTP** **Pru p 3**	(21, 29, 31)

Linoleinic acid [C18:3 cis-9,12,15]	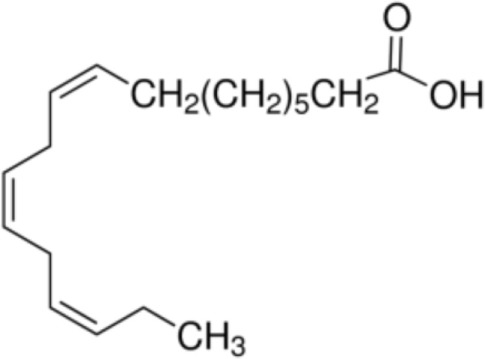	**Pea LTP** (Tri a 14) (Maize LTP)	(21) (29) (14)

**(Phospho) Lipids**			

LLPC [C12]	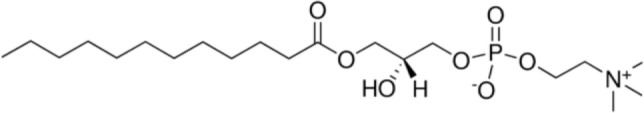	Barley LTP	(21)

LMPG [C14]	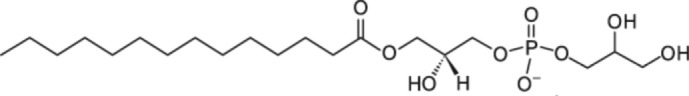	Pea LTP	(21)

LMPC [C14]	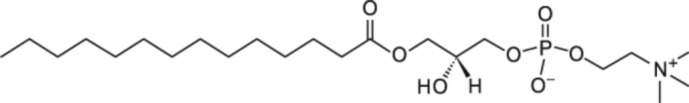	**Wheat LTP1** **Pea LTP**	(20) (14)

DMPG [C14/C14]	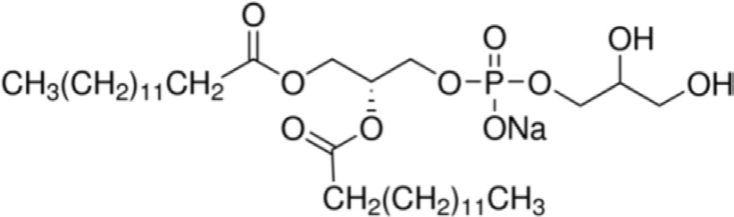	**Wheat LTP1**	(20)

LPPG [C16]	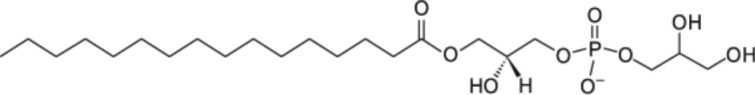	**Wheat LTP2** **Pea LTP**	(23) (21)

LPPC [C16]	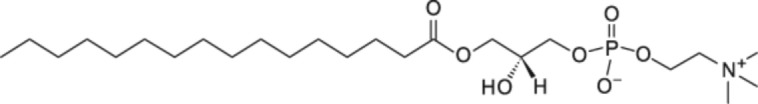	**Tomato LTP** **Pea LTP**	(22) (21)

Phosphatidylcholin	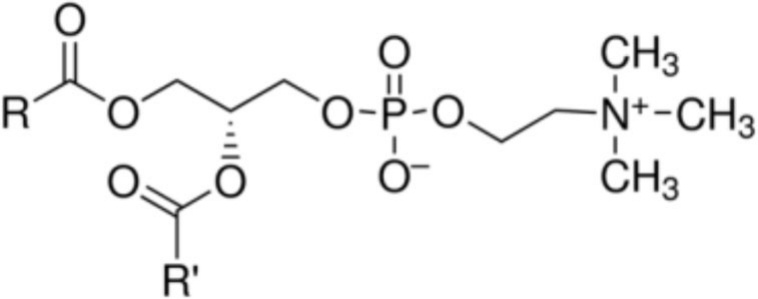	Grape LTP (admixed) **Maize LTP > wheat LTP**	(30) (34)

**Others**			

Phytosphingosine [C18]	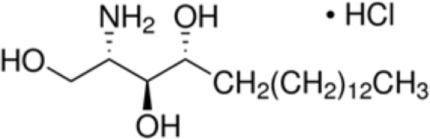	**Pru p 3**	(33)

Prostaglandin B2	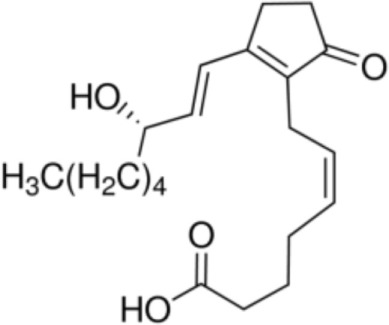	**Wheat LTP1**	(14)

(Ergo)sterol	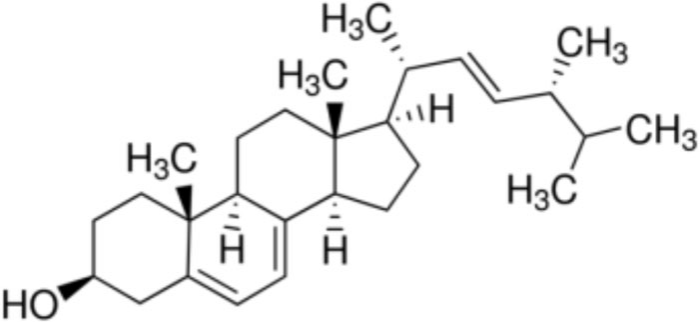	**LTP2** **Rice LTP2**	(19, 24) (24)

Jasmonic acid	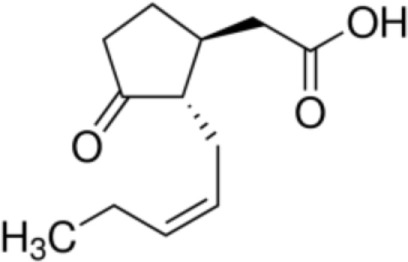	Pea LTP	(21)

α-GalCer	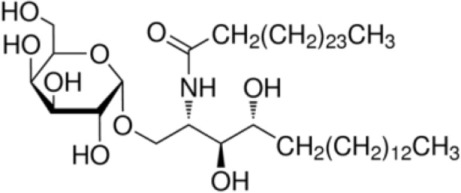	CD1d ligand (control)	(35)

*Chemical structure of lipids and nsLTPs reported to bind the respective lipids are indicated. nsLTPs with strongest lipid binding capacity are indicated in bold, allergenic nsLTPs are indicated according to nomenclature suggested by the IUIS allergen nomenclature subcommittee*.

*LLPC, 1-lauroyl-2-hydroxy-sn-glycero-3-phosphocholine; LMPG, 1-myristoyl-2-hydroxy-sn-glycero-3-phospho-(1′-rac-glycerol); LMPC, 1-myristoyl-2-hydroxy-sn-glycero-3-phosphocholine; DMPG, 1,2-dimyristoyl-sn-glycero-3-phospho-(1′-rac-glycerol); LPPG, 1-palmitoyl-2-hydroxy-sn-glycero-3-phospho-(1′-rac-glycerol); LPPC, 1-myristoyl-2-hydroxy-sn-glycero-3-phosphocholine*.

*nsLTPs, non-specific lipid-transfer proteins; LPPG, L-α-palmitoyl-phosphatidylglycerol; LPPC, L-α-palmitoyl-lysophosphatidylcholine*.

## Interaction of Lipids with Allergenic Food nsLTPs and its Effect on the Allergic Sensitization

Apart from the effect of lipid binding on the stability and IgE-reactivity of nsLTPs, the interaction with lipids might increase immunogenicity of the antigen. Although lipid binding is a common feature of many allergens (6), studies addressing the molecular mechanism by which the innate and allergen-specific adaptive immune responses are triggered are limited.

**Figure 1:**
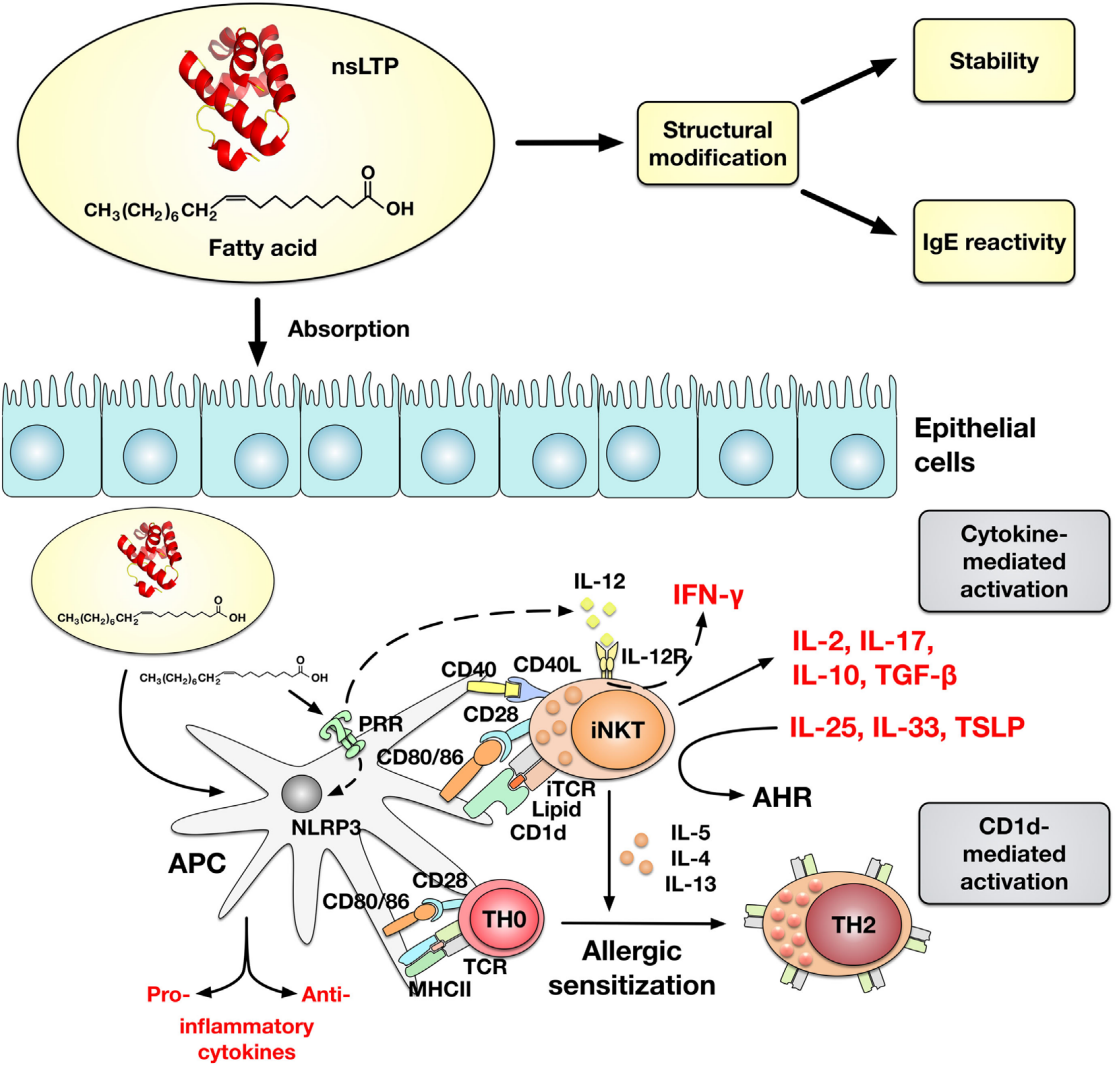
Impact of lipid binding to non-specific lipid-transfer proteins (nsLTPs) on the IgE-reactivity and allergic sensitization. Lipid binding can affect gastrointestinal stability and IgE-reactivity of nsLTPs. Whether the altered IgE-reactivity upon lipid binding is accompanied by structural modification of the protein remains to be elucidated. Moreover, lipids can induce a cytokine- or CD1d-mediated activation of invariant natural killer T (iNKT) cells. Microbial lipids are sensed by pathogen recognition receptors (PRRs) on APCs (DC and Mϕ) and induce an IL-12-mediated activation of iNKT cells which is accompanied by INF-γ secretion. Other lipid ligands which are administrated to APCs in a complex with nsLTPs are internalized, loaded on CD1d, and presented to the TCR (Vα24/Jα18^+^ in humans) on iNKT cells. The CD1d-restricted activation of iNKT cells by nsLTP–lipid complexes is suggested to promote the allergic sensitization by inducing T_H_2-cytokine secretion. In line with this, iNKT cells can promote airway hyperreactivity (AHR) in the presence of IL-25, IL-33, and TSLP. However, activated iNKT cells can display a T_H_1- (INF-γ) and T_H_2- (IL-5, IL-4, and IL-13) like plasticity and lipids can either promote or attenuate NLRP3 inflammasome activity and induce both pro- or anti-inflammatory cytokines.

Recently, it has been reported that an olive pollen-derived lipid fraction was capable to upregulate CD1d (a class I MHC-like molecule) expression on human macrophages (Mϕ) and moDCs, and to activate invariant natural killer T (iNKT) cells (36, 37). Although an allergen-specific response was not investigated, the authors suggested lipid binding to the allergen to have an influence on the allergen-specific immune response. Experimental evidence was provided that binding of a natural lipid ligand to Pru p 3 provokes the allergic sensitization process by involvement of CD1d-mediated activation of iNKT cells (38). The suggested ligand of Pru p 3, 10-OH-camptothecin-phytosphingosine, was presented *via* CD1d on antigen-presenting cells to interact with human (and mouse) iNKTs and was shown to act as an adjuvant to promote IgE-sensitization to Pru p 3 (Figure 1). Notably, α-GalCer, which is well known to induce type I iNKT cell activation, comprises a phytosphingosine, which is an important component for binding to CD1d (35, 39). Interestingly, the complex, but also the lipid ligand alone, induced maturation of human moDCs and proliferation of PBMCs, and its component phytosphingosine was capable to activate NF-kB signaling in the human Mϕ cell line THP-1 (35). By contrast, another study showed the anti-inflammatory activity of phytosphingosine by inhibiting the NF-kB pathway (40). Remarkably, epicutaneous sensitization of BALB/c mice by the Pru p 3-ligand complex induced higher levels of Pru p 3-specific IgE antibodies and enhanced basophil activation in comparison to Pru p 3 alone (38). The authors suggested (1) Pru p 3 to act as carrier and to mimic endogenous saposins which are involved in the loading of lipids to CD1d and (2) the intrinsic adjuvant activity of the accompanying lipid cargo could be a general and essential feature of the mechanism underlying the phenomenon of nsLTP-mediated allergy. Of note, a modification of the lipid cargo phytosphingosine chain can manipulate the iNKT cells to produce different amounts of either IL-4 or IFN-γ polarizing immune response toward either T_H_1 or T_H_2 (35).

In the study by Tordesillas and co-workers (38), the adjuvant effect of lipid alone in regard to its effects *in vivo*, its properties to modulate cytokine secretion in PBMCs, and the CD1d-mediated lipid presentation without Pru p 3 as carrier were not addressed. Although, the interaction of lipids with nsLTPs likely affects the development of an allergic inflammation, it remains to be elucidated whether lipid binding into the cavity of nsLTPs is required to exert all immune-modulating properties. So far, the effect of other described lipid ligands on the allergen-specific immune response to nsLTPs is poorly characterized and it remains unknown whether FA ligands of Pru p 3 have immunological properties to activate iNKT cells.

## Discussion

Non-specific lipid-transfer proteins can bind a broad variety of lipids due to versatile binding abilities of the proteins. Whether the lack of binding specificity can be explained with a structural flexibility of the binding cavity which accounts for the variable lipid-binding modalities between different nsLTPs and isoforms is controversially discussed (41). Moreover, the lipid binding capacity of nsLTPs depends on the chemical nature of the ligand and key amino acids at the entrance of the hydrophobic cavity. The interaction of certain lipids with nsLTPs has been suggested to affect both the IgE-reactivity and immunogenicity of the respective allergens. The hypothesis is deduced from reports that lipid binding to nsLTPs can slightly alter protein structure and therefore also conformational IgE-binding sites affecting the stability and IgE-binding properties of nsLTPs. However, whether lipid binding to nsLTPs increases the intrinsic immunogenicity, by inducing biologically relevant structural changes in the resulting nsLPT:lipid complex and/or by activation the innate immune system is not fully clarified at the moment.

In general, precise analysis of the nsLTP–lipid complex is required to discriminate whether lipids are bound to the cavity of nsLTPs or are simply attached to the surface of the protein. Moreover, for the assessment of lipid binding properties of nsLTPs any natural, pre-loaded lipids need to be excluded. This is of particular importance since the purification of recombinant nsLTPs can be associated with a co-purification and binding of lipids derived from the natural source or the applied heterologous expression system (25). Finally, it should be taken into account that the composition of lipids and free sterols changes due to development and ripening of fruits (42).

Whereas the mode of interaction between lipids and nsLTPs is well investigated, the effects of lipid binding on the allergic sensitization and the molecular mechanisms in effector cells are largely unknown. In line with this, it could also be speculated that nsLTP interaction with lipids will protect from gastrointestinal digestion and promote the absorption of intact nsLTPs into the intestinal tissue and blood stream. Although not experimentally proven, the interaction of nsLTPs with the lipid bilayer of the cell membrane might mediate a facilitated endocytosis and subsequent antigen processing and presentation.

The interaction or co-exposure of lipids together with nsLTPs is an attractive model to further explain the pathomechanism of nsLTP-mediated allergies, but also other hypersensitivity reactions triggered by allergens with lipid-binding properties. Here, the suggested function of nsLTPs as lipid carriers, e.g., to target lipid adjuvants and antigen simultaneously to APCs, needs to be further investigated. So far, lipid binding to nsLTPs has been shown to result in CD1d-restricted activation of iNKT cells promoting allergic sensitization (Figure 1). However, additional mechanisms, likewise lipids engaging pathogen recognition receptors (PRRs) and lipids influencing the absorption of allergens through the epithelial barrier, might be involved to modulate the immunogenicity of nsLTP:lipid complexes (43). Future research needs to consider that nsLTP-associated lipids potentially can function as danger signals to activate PRRs and can influence the NLRP3 inflammasome in APCs. Specifically, saturated FAs have been shown to promote inflammation and polyunsaturated FAs have been shown to impede inflammasome activity (44). Whether FA delivered to APCs by nsLTP cargo proteins are be capable to modulate inflammasome activity has not been investigated. Taking into account the diversity of lipid ligands, it could be speculated that both pro- or anti-inflammatory immune responses and T_H_1- or T_H_2-polarized antigen-specific immune responses could be induced, respectively. In fact, lipids possess intrinsic adjuvant activity and likely can modulate the allergenicity of proteins not only by altering protein structure and stability upon binding (29) but also by direct interaction of bound and unbound lipids with immune cells. Free FA can modulate proliferation and cytokine secretion of T cells differentially, depending on their saturation grade and the stimuli used (43).

The reported studies describing lipid binding to nsLTPs have used different experimental approaches to tackle the complex task of characterizing lipid binding to nsLTPs: (1) *in silico* experiments by molecular modeling have been applied to predict interactions between different lipid ligands and the binding cavity of the respective nsLTP, (2) purified nsLTPs have been exposed to different lipids *in vitro* to determine the binding capacity of the respective lipids to the tested nsLTP and finally, and (3) lipids ligands were isolated from natural Pru p 3 in order to identify the lipids naturally bound to the allergen.

While *in vitro* binding experiments have shown that, in accordance with their function as nsLTPs, the different nsLTPs tested can bind a variety of different lipids, unexpectedly the lipid isolation experiments using natural purified Pru p 3 reported a single, major lipid ligand to be bound to the cavity of Pru p 3. Here, future research should aim to systematically characterize ligand binding to both allergenic- and non-allergenic LTPs. It is tempting to speculate that lipid binding and/or the activation of the innate immune system lipids contributes to the higher allergenicity of certain nsLTPs.

## Author Contributions

Both authors contributed equally to the manuscript.

## Conflict of Interest Statement

The authors declare that the submitted work was not carried out in the presence of any personal, professional, or financial relationships that could potentially be construed as a conflict of interest.
